# Genome Sequence of “*Candidatus* Viridilinea halotolerans” Chok-6, Isolated from a Saline Sulfide-Rich Spring

**DOI:** 10.1128/MRA.01614-18

**Published:** 2019-01-24

**Authors:** Denis S. Grouzdev, Ekaterina I. Burganskaya, Maria S. Krutkina, Marina V. Sukhacheva, Vladimir M. Gorlenko

**Affiliations:** a Research Center of Biotechnology, Russian Academy of Sciences, Moscow, Russia; Queens College

## Abstract

The draft genome sequence of the green filamentous anoxygenic phototrophic (FAP) bacterium “*Candidatus* Viridilinea halotolerans” strain Chok-6, isolated from a cold saline sulfide-rich spring near Lake Chokrak, is presented. The genome sequence is annotated for elucidation of the taxonomic position of Chok-6 and to extend the public genome database.

## ANNOUNCEMENT

All known mesophilic green filamentous anoxygenic phototrophic (FAP) bacteria belong to suborder *Chloroflexineae* (1). With the exception of Oscillochloris trichoides (2–4), mesophilic green FAP bacteria “*Candidatus* Chlorothrix halophila” (5), “*Candidatus* Chloroploca asiatica” (6), and “*Candidatus* Viridilinea mediisalina” (7) are available only as enrichment cultures. Despite this limitation, the development of metagenomics methods allows for the reconstruction of the genome sequences of these bacteria (8, 9).

The mesophilic green FAP strain Chok-6 was isolated from a saline sulfide-rich Chokrak spring (22 g liter^−1^ NaCl), located on the northeastern coast of the hypersaline Lake Chokrak (lat 45.46, long 36.31). Chok-6 was isolated in a stable enrichment culture and maintained at 25 to 35°C in light (2000 lx) using the previously described medium (7) with the following modifications (g liter^−1^): NaCl, 5.0; Na_2_S·9H_2_O, 0.5; and NaHCO_3_, 3 (without Na_2_S_2_O_3_). The pH value of the medium was 7.5. Chok-6 was isolated from a brown-green microbial mat and had bacteriochlorophyll *c* (749 nm) as the main photosynthetic pigment. The wavelength of the pigments was determined in a 50% glycerol cell suspension with a SF-56a spectrophotometer (OKB Spectr).

Genomic DNA was extracted using a DNeasy PowerSoil kit (Qiagen) according to the manufacturer’s instructions. Libraries were constructed with the NEBNext DNA library prep reagent set for Illumina, per the kit’s protocol. Sequencing was undertaken with the Illumina HiSeq 1500 platform with pair-end 230-bp reads. A total of 4,884,260 reads were obtained from Chok-6. Raw reads were quality checked with FastQC v. 0.11.7 (http://www.bioinformatics.babraham.ac.uk/projects/fastqc/), and low-quality reads were trimmed with Trimmomatic v. 0.36 (10). Trimmed reads for all samples were assembled using metaSPAdes v. 3.12.1 (11) at the default settings. Metagenome binning was performed using three binning algorithms, BusyBee Web (12), MaxBin 2.0 2.2.4 (13), and MyCC (14). The three bin sets were supplied to DAS Tool 1.0 (15) for consensus binning to obtain the final optimized bins. Genome bins were assessed for completeness and contamination with CheckM 1.0.11 (16). The final assembled 6,104,039-bp-long genome comprised 972 scaffolds, with an *N*_50_ value of 9,167 bp, an average coverage of 32×, and a GC content of 60.4%. Annotations of the scaffolds were carried out with the NCBI Prokaryotic Genome Annotation Pipeline (17), which identified 4,725 genes, 4,670 coding sequences, 149 pseudogenes, and 45 tRNA genes. The average nucleotide identity (ANI) (18) and digital DNA-DNA hybridization (dDDH) (19) values of 81.3% and 27.6%, respectively, to the genome of the closest strain, “*Ca.* Viridilinea mediisalina” Kir15-3F, were below the criteria for assignment to separate species (20), which indicates that the strain Chok-6 belongs to a new *Viridilinea* species with the proposed name “*Candidatus* Viridilinea halotolerans.” The genome sequence of “*Ca.* Viridilinea halotolerans” contains all the necessary genes for bacteriochlorophyll *a*, *d*, and *c* biosynthesis, including those absent from Oscillochloris trichoides *acsF* and absent from members of the genus *Chloroflexus bchQ* and *bchR* (21). *NifHBDK* nitrogen fixation genes are present, but *nifEN* and *nifV* are absent. Among FAP bacteria, besides representatives of *Viridilinea*, a similar gene cluster is present in representatives of the genera *Roseiflexus* and *Oscillochloris*. In addition, “*Ca.* Viridilinea halotolerans” has the genes for the 3-hydroxypropionate cycle of the autotrophic system for assimilating CO_2_. The genome sequence lacks genes of the *sox* system for thiosulfate oxidation, but it contains the gene of sulfide:quinone oxidoreductase for sulfide oxidation.

### Data availability.

This whole-genome shotgun project has been deposited in DDBJ/ENA/GenBank under the accession no. RSAS00000000. The version described in this paper is the first version, RSAS01000000. The raw FASTQ reads have been deposited in the NCBI SRA database under the accession no. SRR8257186.
